# Understanding premarital pregnancies among adolescents and young women in Ouagadougou, Burkina Faso

**DOI:** 10.1080/23311886.2018.1514688

**Published:** 2018-09-25

**Authors:** Abdramane Bassiahi Soura, Yempabou Bruno Lankoande, Souleymane Sanogo, Yacouba Compaore, Leigh Senderowicz

**Affiliations:** 1 Institut Superieur des Sciences de la Population (ISSP), University Ouaga 1 Pr Joseph Ki-Zerbo, Ouagadougou, Burkina Faso; 2 Université Catholique de Louvain, Louvain-la-Neuve, Belgium; 3 Department of Global Health and Population, Harvard University, Boston, USA; 4 University of Sydney, Australia

**Keywords:** adolescents, young women, pregnancy, premarital, Ouagadougou

## Abstract

In developing countries, young women between 15 and 24 years of age account for more than 40% of unsafe abortions due to the high number of unwanted and/or out-of-wedlock pregnancies. However, much about the profile of adolescents and young women who usually experience premarital pregnancies remains unknown. This study sought to understand the risk of pregnancy before marriage among adolescents and young women in Ouagadougou, Burkina Faso. By using longitudinal data from a demographic surveillance system, we tested the explanatory power of two theoretical assumptions on premarital childbearing in sub-Saharan Africa, which assumptions are the cultural inheritance model and the social capital model. The results confirmed the explanatory power of the cultural inheritance model on the one hand and partially confirmed the power of the social capital model on the other hand. These results highlight the need for a multipronged approach to sexual and reproductive health for young people. Efforts against premarital pregnancies among adolescents and young women would be more effective if they were based on participatory approaches, incorporating actions at both community and institutional levels, as suggested by the recent Global Accelerated Action for the Health of Adolescents logical framework.

## PUBLIC INTEREST STATEMENT

Pregnancies among adolescents and young women before marriage are a major issue in Burkina Faso. The possible consequences of such pregnancies are multiple, ranging from girls leaving school early, to their family and social exclusion, unsafe clandestine abortions, child abandonment, contested paternity, and difficult living conditions.However, much about the profile of adolescents and young women who usually experience premarital pregnancies remains unknown. This study sought to identify factors influencing premarital pregnancies among women aged 15-24 years in Ouagadougou, Burkina Faso. The empirical results showed that religion, ethnic group and family characteristics were the most important determinants. These results call for a multipronged approach to sexual and reproductive health for young people, which includes combating poverty, strengthening sexual education, and improving access to contraceptives. They also suggest that, sexual education should not be solely carried by educators and/or specific programs but, should also involve parents more than any other actor.

## 1. Introduction

A large number of studies in sub-Saharan Africa showed that premarital sexual activity is increasing, and more and more occurring at an early age in many countries (Gupta & Mahy, 2003; Mensch, Grant, & Blanc, 2006; UNICEF, 2015; Wellings et al., 2006). Because contraceptive prevalence remains low in this region (Khan & Mishra, 2008; Lesthaeghe, 2014), premarital sexuality is often associated with early motherhood and/or abortion (Neal, Chandra-Mouli, & Chou, 2015; Zabin & Kiragu, 1998). Findings from Sedgh et al. (2016) showed that between 1990 and 2014, approximately 86% of all abortions worldwide occurred in low- and middle-income countries, and the most recent data disaggregated by age showed that in 2008, young women aged 15–24 years accounted for 41% of all unsafe abortions in these countries (Shah & Åhman, 2012). Early childbearing may negatively affect maternal and child health, while out-of-wedlock pregnancies may have adverse socioeconomic outcomes (Adjamagbo et Kone, 2013; Karra & Lee, 2012). For instance, a premarital pregnancy may force a young mother to temporarily or permanently interrupt her schooling. If the premarital pregnancy is unwanted, it may still have serious consequences for the child’s living conditions (Adjamagbo et Kone, 2013). Moreover, findings from Calves (2000) showed that in some African societies, children born out of wedlock are exposed to certain forms of discrimination.

Currently, much remains unknown about the profile of adolescents and young women who experience pregnancies out of wedlock. This paper aims at reporting findings from a study that sought to identify some explanatory factors of premarital pregnancies among adolescents and young women in Ouagadougou, the capital city of Burkina Faso. The study considered different variables related to the woman’s family and her individual characteristics. It used longitudinal data from a demographic surveillance system, which registered all vital events (pregnancies, births, marriages, migrations) that occurred in the life of individuals over time.

Burkina Faso is one of the poorest countries in the world (UNDP, 2016), located in the Sahel region of West Africa. Our study took place in a city of 2.7 million inhabitants (UNITED NATIONS, 2014), where education on sexuality is weak (Rossier, Sawadogo, & Soubeiga, 2013) and contraceptive prevalence low. For instance, in the Ouagadougou Health and Demographic Surveillance System (HDSS), a 2010 health survey showed that, among unmarried women with a need for contraception (women who do not wish to have a child in the next 2 years), only 23% used a modern method of contraception (Rossier & Ortiz, 2011).

## 2. Theoretical background

Though several previous works explored the levels and consequences of premarital fertility in sub-Saharan Africa (Clark, Koski, & Smith-Greenaway, 2017; Garenne & Zwang, 2008; Meekers, 1994; Parr, 1995; Zwang & Garenne, 2008), the explanatory factors remain unclear. Cherlin and Riley (1986) were among the first scientists to explain premarital childbearing of African adolescents, by proposing two models: the rational adaptation model and the social disorganization model. The rational adaptation approach argues that the woman’s decision to engage in a sexual activity is a carefully considered one, based for example on the expectation of some sort of advantage or gain. According to this model, a woman living in poverty might seek an advantageous marriage by demonstrating her fertility, or creating stronger, more binding ties to her partner through a pregnancy.

The social disorganization model on the contrary rests on the assumption that sexual activities before marriage and out-of-wedlock fertility are “accidental” (Cherlin & Riley, 1986). It explains premarital childbearing as a result of the weakening of social control and the adoption of more liberal attitudes that come with modernization. Following Cherlin and Riley (1986), a synthesis presented by Emina (2009) proposed three other models of premarital fertility in sub-Saharan Africa: (1) the demographic model, (2) the cultural inheritance model, and (3) the social capital model. The demographic model attributes premarital childbearing to two factors: a low contraceptive prevalence (Boohene, Tsodzai, Hardee-Cleaveland, Weir, & Janowitz, 1991; Garenne, Tollman, & Kahn, 2000) and a growing gap between puberty and first union that prolongs the woman’s period of exposure (Reda & Lindstrom, 2014).

The cultural inheritance model posits that cultural differences regarding fertility practices cause different outcomes in premarital fertility (Suset, 2005; Zhang, Poston, Alvard, & Cherry, 2013). According to this model, reproductive behaviors result (either directly or indirectly) from the intergenerational transmission of traditional and normative values (such as those conveyed by ethnicity and religion) surrounding procreation. As a result, cultural inheritance may be a contributing factor to earlier or later fertility, depending on whether traditional cultural norms and values accept or punish sex and procreation before marriage (Emina, 2009; Gage, 1998).

The social capital model is close to the rational adaptation argument, recognizing the rationality of individuals. However, it also considers that each person lives in a social context that will influence his/her sexual behavior (Coleman, 1988; Laumann, Gagnon, Michael, & Michaels, 1994). At the micro level, Emina (2009) put forward three explanatory factors of premarital childbearing: the household’s standard of living, human capital, and the size and composition of the household. Women from poor households are the most vulnerable to premarital pregnancies. In these households, access to health services and contraceptive methods may be limited due to a lack of financial means. Moreover, the influence of the family environment is essential, especially the ability of parents/guardians to communicate with their children. A lack of communication between parents and children about sexuality can expose young people to risky sexual behaviors. The educational level of the head of household appears to be a good proxy for identifying the effect of human capital, with the idea that well-educated parents are better able to converse with their children about sexuality (Adjamagbo, Antoine, & Delaunay, 2004; Akam & Arroga, 1998). The size and the composition of the household also may influence premarital sexuality through the absence of close supervision of the children (Emina, 2009). Indeed, although the ability to manage household members depends on the standard of living and the human capital, the attention given to each child may depend on the number of dependents and their relationship with the head of the household.

There is scant research attempting to test the explanatory power of the abovementioned theories in sub-Saharan Africa, perhaps because of the scarcity of data. In our study, we were interested in testing the explanatory power of two of these theories, that is, the social capital and the cultural inheritance models. The three other theories (demographic, rational adaptation, and social disorganization models) could not be sufficiently assessed because of insufficient availability of relevant information. Thus, they were not the main focus of our study even if some control variables may have reflected their influence.

We tested the cultural inheritance model through the influence of ethnicity and religion. Some ethnic groups in Burkina Faso, such as the Mossi, are generally thought to hold more conservative ideas in terms of sexuality than other Burkinabe ethnic groups (Bonnet, 1988; Taverne, 1999). Likewise, there is evidence that sexual behavior can be tied to religious precepts (Adjamagbo et al., 2004). Islam and Christianity, the two dominant religions in Ouagadougou, are both restrictive in terms of premarital sexuality (Dialmy, 2010; Thornton & Camburn, 1989), but in the Burkinabe context, Christianity is more associated with Western culture and a certain cultural openness, which are in turn correlated to premarital sexual activity. In connection with the cultural inheritance model, we tested two hypotheses: a lower risk of premarital pregnancy among the Mossi women compared to the other ethnic groups (Hypothesis 1), and a higher risk of premarital pregnancy among Christian women, compared to Muslim women or women of other religions (Hypothesis 2).

Regarding the social capital model, we were interested in the influence of the living standard of the household, the size of the household, the educational level of the head of household, and the parental presence in the household. We also added a variable for the type of neighborhood, which is particularly salient in the Ouagalese context. Like in many African cities, Ouagadougou consists of both formal neighborhoods and informal neighborhoods. Formal neighborhoods are well structured, with full access to municipal services such as electricity, water, and sanitation, while informal neighborhoods are mainly unplanned settlements that sprang up as a result of a rapid urbanization (Soura, 2014). Their inhabitants are, according to a study conducted by Rossier, Soura, Lankoande, and Millogo (2011), often poorer and less educated than their formal neighborhood counterparts, which means lower human capital in the informal settlements. This poor standard of living often means a lack of health facilities, including reproductive health services. Consequently, the contraceptive prevalence is lower in informal neighborhoods than in the formal ones, as suggested by findings from Rossier and Ortiz (2011). Considering this, we tested the assumption that adolescents and young women in informal neighborhoods are at a higher risk of becoming pregnant before marriage than in formal neighborhoods (Hypothesis 3). We also tested a higher risk of getting pregnant before marriage among adolescents and young women living in poorer households (Hypothesis 4) as well as in larger households (Hypothesis 5) and in households headed by less educated persons (Hypothesis 6). We additionally tested the hypothesis that parental presence in the household is associated with a lower risk of premarital pregnancy (Hypothesis 7).

## 3. Methodology

### 3.1. Data

We used longitudinal quantitative data from the Ouagadougou HDSS, gathered between 2009 and 2015. The Ouagadougou HDSS is a platform for research and interventions set up in 2008 in five districts of Ouagadougou (Rossier et al., 2012). Two of these districts are formal neighborhoods (Kilwin, Tanghin) while the other three (Nonghin, Polesgo, Nioko) are informal settlements. Ethical approval for the Ouagadougou HDSS was granted by the Ethics Committee for Health Research of the Ministry of Health of Burkina Faso.

After an initial census conducted between October 2008 and March 2009 in the study site, fieldworkers have conducted periodic household update rounds, registering pregnancies, births, deaths, marriages, and migrations with an average periodicity of 10 months. In addition to these routine rounds, researchers also conducted occasional surveys on health or urban poverty to gather more specific and detailed information on these topics of interest. The data collected constitute a longitudinal database for measuring changes over time in the population or testing interventions. As of December 2015, the Ouagadougou HDSS database contained over 90,000 inhabitants, of which 53% lived in the informal areas and 47% in the formal ones.

Our dataset included 10,022 single women aged 15–24, who were surveyed as residents (defined as individuals present in the zone for at least 6 months) in the HDSS. Those 10,022 women were all those who had at least one episode of residence in the HDSS over the study period (between January 2009 and December 2015), were single, and aged 15–24. Because of left and right censoring in such longitudinal data, not all episodes of residence had the same exposure time. Table 1 explains how exposure was calculated as a function of entry type and exit type. The start date for each subject was 01 January 2009 or its date of immigration, whichever was later. In the Ouagadougou HDSS, the date of the pregnancy onset is estimated by adding 14 days to the date of the last menstruation.

**Table 1.** Exposure calculation as a function of the entry type (in line) and the exit type (in column) between 01 January 2009 and 31 December 2015

	Exit by pregnancy before 31 December 2015	Exit by marriage before 31 December 2015	Woman turning 25 during the follow-up period	Exit by out-migration	Exit at the end of observation (31 December 2015)
Woman in the 15–24 age group before the start date	Difference between the start date and the onset date of the pregnancy	Difference between the start date and the date of marriage	Difference between the start date and 25th birthday	Difference between the start date and the date of out-migration	Difference between the start date and 31 December 2015
Woman turning 15 after the start date	Difference between 15th birthday and the onset date of the pregnancy	Difference between 15th birthday and the date of marriage	N/A (not possible in 6 years of follow-up)	Difference between 15th birthday and the date of out-migration	Difference between 15th birthday and 31 December 2015

In this study, the sum of the exposures was converted into person-years and used as the denominator in the calculation of the rate of a first premarital pregnancy. The 10,022 women totaled 54,078.2 person-years at risk between January 2009 and December 2015.

### 3.2. Variables

#### 3.2.1. Dependent variable

The dependent variable in our study was the occurrence of a first pregnancy before marriage. We used a working definition of marriage that included the union between two persons of opposite sex, whether this union was created by a religious, customary or civil ceremony, as well as cohabitation. Our pregnancy variable was dichotomous, taking the value of 1 in case of a premarital pregnancy and 0 otherwise.

#### 3.2.2. Independent variables

Our main independent variables included religion, ethnic group, standard of living, type of neighborhood, educational level of the head of household, size of the household, and parental presence in the household. To these main independent variables (and in accordance with the theoretical background), we were able to add two control variables: the woman’s educational level and her age group.

In Burkina Faso, there are about 60 different tribes classified into 11 ethnic groups that are spread across the country (Giordan, 2014). The Mossi, who make up the country’s largest ethnic group (Giordan, 2014), were also the majority group in our study site, representing over 90% of our population. Other ethnic groups (Fulani, Tuareg, Lobi, Senufo, Bissa, Bwa, Bobo, Gourmantche, Gourounsi, Samo) were distributed in much smaller proportions (less than 2% each). For the purposes of our analysis, the ethnic group was coded into two categories: the Mossi and the Others.

Generally speaking, Islam and Christianity are the two dominant religions in Ouagadougou. A previous research conducted in the study site by Rossier et al. (2011) showed that 61% of the residents were Muslim and nearly 39% Christian. Animists and people without religion represented less than 1% of the population (Rossier et al., 2011). In this paper, we have grouped Muslims and animists together in order to avoid the problem of small numbers and make it possible to test our Hypothesis 2.

A lack of data on direct income led the Ouagadougou HDSS team to create a proxy variable for a standard of living that considered both the presence of a refrigerator and a television in the household, as well as the most expensive mode of transport available in the household (Soura, Pison, Senderowicz, & Rossier, 2013). A household with both a motorcycle and a car, for example, was classified as a car-owning household. The coefficient attached to each good was derived from a principal components analysis using data collected during the first round. Households were grouped into three categories (the poorest, the middle class, and the wealthiest), which were updated annually.

This standard-of-living approximation based on household assets is often done by demographers but is not exempt from criticism. Montgomery, Gragnolati, Burke, and Paredes (2000) showed in an analysis of five developing countries that living standard’s proxies based on the housing characteristics and assets do not always have a strong correlation with household expenditures. However, as they argued, “demographers are fortunate in having access to relatively large samples, and sample size further enhances the power of the proxy-based test” (Montgomery et al., 2000, p.170).

The other main independent variables (type of neighborhood, educational level of the head of household, size of the household, and parental presence in the household) were also time-varying covariates. In accordance with standard practices in demographic surveillance systems (Emina et al., 2011; Ye, Wamukoya, Ezeh, Emina, & Sankoh, 2012), the Ouagadougou HDSS updated the list of household members during each surveillance round, leading to possible changes in heads of households and the presence of parents in households. Thus, a person may had changed neighborhoods during the follow-up; a head of household may had died or left the household and was therefore replaced by another head of household. Similarly, the father or mother of a person may had died or migrated or even changed households. All these events and their dates were collected in the HDSS.

As previously mentioned, there were two types of neighborhoods, namely, the formal and the informal. The educational level of the head of the household was divided into three categories: no education, primary level, and secondary or higher level of education. The size of the household was calculated based on the number of people living in the household. It varied from 1 to 40 but based on its distribution, we made four groups for this variable to avoid small numbers, that is, less than or equal to 4 people, 5–6 people, 7–8 people, and 9 or more people. With regards to parental presence in the household, we also distinguished four categories, namely, (1) neither of the two parents in the household, (2) presence of the father alone, (3) presence of the mother alone, and (4) presence of both parents in the household.

The two control variables added to the main independent variables were the woman’s age group and her educational level. Each variable was divided into two categories in order to avoid the problem of small numbers in some categories. For the age group, we distinguished women of 15–19 years old from those of 20–24, and for the educational level, we also distinguished the primary level or below from the secondary level or higher.

The sample’s characteristics are presented in Table 2.

**Table 2.** Characteristics of the sample

Variables	Persons-years	Percentage
**Age group**		
15–19 years	28,594.1	52.9
20–24 years	25,484.1	47.1
**Religion**		
Christian	21,984.8	40.7
Muslim and other	32,093.4	59.3
**Ethnic group**		
Mossi	49,056.2	90.7
Other	5,022	9.3
**Education**		
Primary or less	33,032.7	61.1
Secondary or high	21,045.5	38.9
**Standard living**		
Low	14,405	26.6
Medium	29,396.8	54.4
High	10,276.4	19.0
**Education of H. head**		
No education	16,944.8	31.3
Primary	19,337.5	35.8
Secondary or high	17,795.9	32.9
**Household size**		
≤ 4	9,010.8	16.7
5–6	16,549.3	30.6
7–8	12,826.1	23.7
≥9	15,692	29.0
**Parental presence**		
Neither	5,289.6	9.8
Father only	1,978.6	3.7
Mother only	7,007.1	13.0
Both parents	39,802.9	73.6
**Type of area**		
Informal	20,881.1	38.6
Formal	33,197.1	61.4
**Total**	54,078.2	100.0

The majority of women in our sample were Mossi. Most women were also Muslim and had some primary education or below. Those women were, on average, slightly more educated than the heads of their households. They mostly lived in households with five people or over. Regarding the standard of living, the middle class was the most represented. Women between 15 and 19 years of age were more represented in this sample, and as to the parental presence, about three out of four lived with both parents, and commonly in formal settlements.

### 3.3. Statistical analysis

Our statistical analysis included a bivariate descriptive component as well as a multivariate component. Data were organized to accommodate the calculation of pregnancy rate, which was obtained through the division of the number of pregnancies by the person-years exposed in each subpopulation. The pregnancy rates were produced by the Stata software (version 14) with the associated 95% confidence intervals. These confidence intervals were used to compare rates in terms of statistical differences. In principle, if two rates have overlapping confidence intervals, it cannot be said that the corresponding categories (or subpopulations) have different premarital pregnancy rates. If the confidence intervals do not overlap, it means that the two corresponding categories have different premarital pregnancy rates.

Cox regression was used for the multivariate analysis. The Cox model tested whether the hazard of becoming pregnant before marriage differed from one group to another, after controlling for possible confounding variables. By principle, this model relies on the proportional hazards assumption, which postulates that the relationship of hazards between individuals in two different groups does not vary over time (in this study, the length of stay in the HDSS area). In general, this assumption weakens (or even becomes untenable) if several covariates change over time (Allison, 1995). In our case, except for ethnicity and religion, all other variables were considered time-varying covariates. Additionally, since the data used here were not sample data, we utilized the bootstrap method with 100 replications to get robust confidence intervals (Salibian-Barrera & Zamar, 2002).

With respect to the interpretation, for each given variable, the output of the Cox regression is a hazard ratio compared to a reference category. The hazard ratio of the reference category is by definition equals to 1. In our analysis, if the hazard ratio was significantly greater than 1, then the risk of getting pregnant before marriage was higher in the corresponding category compared to the reference category. The opposite situation prevailed if the hazard ratio was significantly lower than 1. In Section 4, for each hazard ratio, we indicated by stars the level of statistical significance, which allowed us to conclude that this hazard ratio was different from 1 with a risk of 5% or 1‰ to be mistaken. As it is customary in the social sciences, we accepted a maximum error of 5% to admit that a difference was significant.

The 95% confidence interval associated with each hazard ratio is also contained in Section 4. The comparison of these confidence intervals between two categories made it possible to say if there was any significant difference or not between these categories in terms of getting pregnant before marriage, all other things being equal.

## 4. Results

### 4.1. Descriptive results

Descriptive results are presented in Table 3. The rate of experiencing a premarital pregnancy among adolescents and young women (15–24 years old) was estimated at 7.30 per 1,000 person-years. Out of the five variables used to test the explanatory power of the social capital model, four had a significant relationship with the risk of a premarital pregnancy: the standard of living of the household, the level of education of the head of household, the parental presence in the household and the type of neighborhood in which the family lives. For each of these variables, there were at least two categories that had different significant rates.

**Table 3.** Premarital pregnancy rates

Variables	Rates (p. 1,000 person-years)	95% Conf. interval	
**Age group**			
15–19 years	4.0	3.3	4.8
20–24 years	11.1	9.8	12.4
**Religion**			
Christian	8.3	7.2	9.6
Muslim and other	6.6	5.8	7.6
**Ethnic group**			
Other	9.2	6.9	12.2
Mossi	7.1	6.4	7.9
**Education**			
Primary or less	6.4	5.6	7.3
Secondary or more	8.8	7.6	10.2
**Standard living**			
Low	9.9	8.4	11.7
Medium	7.4	6.5	8.4
High	3.4	2.4	4.7
**Education of H. head**			
No education	10.1	8.6	11.7
Primary	5.3	4.3	6.4
Secondary and plus	6.9	5.7	8.2
**Household size**			
≤4	8.8	7.0	10.3
5–6	6.6	5.5	8.0
7–8	8.0	6.6	9.7
≥9	6.6	5.4	8.0
**Parental presence**			
Neither	3.4	2.1	5.4
Father only	9.6	6.1	15.0
Mother only	8.6	6.6	11.0
Both parents	7.5	6.7	8.4
**Type of area**			
Informal	8.8	7.6	10.1
Formal	6.4	5.6	7.3
**Total**	**7.3**		

Table 3 shows that out of 1,000 unmarried women from poor households who had never been pregnant before, about 10 got pregnant annually, while these rates were lower in women of middle or higher classes, with 7.4 and 3.4 pregnancies per 1,000, respectively. These three rates were significantly different, based on the comparison of their confidence intervals (Table 3). Premarital pregnancy was also more frequent in informal neighborhoods, where the rate was 8.8‰ as compared to 6.4‰ in formal neighborhoods (Table 3). The educational level of the head of household did not appear to have a linear relationship with the woman’s risk of getting pregnant, but our data indicated that the risk was higher if the head of household did not attend school. The rate was estimated at 10.1‰ if the head had no education, as opposed to 5.3‰ and 6.9‰ for the primary and secondary or higher levels, respectively. The two last categories (primary, and secondary or higher) had no significant difference in terms of premarital pregnancy rate (Table 3). A likewise nonlinear relationship was observed with the parental presence in the household. When neither the father nor the mother was present, the risk of getting pregnant was lower, in comparison with the women who had one or both parents (Table 3). A difference was not observed between those who had the father or the mother only, and those with both parents (Table 3).

The relationship with cultural variables was not significant at 95% confidence, neither for the religion nor for the ethnicity (Table 3). Regarding the two control variables (women’s age group and their educational level), it appeared that the pregnancy rate increased more than twice when adolescents aged between 15 and 19 were compared to women between 20 and 24 years old (4.0 pregnancies vs. 11.1 per 1,000 person-years, respectively). In relation with women’s educational level, the difference in becoming pregnant before marriage was small between women with secondary or greater education and women at the primary level or below, though there was a slight tendency for the former to be more exposed to premarital pregnancies (8.8‰ vs. 6.4‰).

### 4.2. Multivariate results

The multivariate findings (Table 4) corroborated much of what was observed in the descriptive results. Three of the five variables related to the social capital theory had a significant relationship with the risk of getting pregnant before marriage. Those were the standard of living of the household, the educational level of the head of household, and the parental presence in the household.

**Table 4.** Hazard ratios of premarital pregnancy (Cox regression)

Variables	Hazard ratios	95% Conf. interval	
**Age group**			
15–19 years	0.37**	0.29	0.46
20–24 years	Ref.		
**Religion**			
Christian	1.28*	1.04	1.57
Muslim and other	Ref.		
**Ethnic group**			
Mossi	0.64**	0.46	0.90
Other	Ref.		
**Education**			
Primary or less	Ref.		
Secondary or high	1.31*	1.02	1.68
**Standard living**			
Low	Ref.		
Medium	0.76*	0.61	0.96
High	0.35**	0.23	0.53
**Education of H. head**			
No education	Ref.		
Primary	0.74*	0.58	0.96
Secondary or high	0.68**	0.51	0.90
**Household size**			
≤4	Ref.		
5–6	0.91	0.67	1.22
7–8	1.13	0.84	1.53
≥9	0.99	0.72	1.37
**Parental presence**			
Neither	Ref.		
Father only	2.4**	1.26	4.60
Mother only	2.2**	1.28	3.72
Both parents	2.2**	1.37	3.60
**Type of area**			
Informal	Ref.		
Formal	0.85	0.67	1.07

Time at risk: 54,078.2; number of failures: 395. LR Chi^2^: 187.6; *p*-value of Chi^2^: 0.000. *p < 0.05; **p < 0.001.

The relationship with the standard of living was negative, with the poorest more likely to experience a premarital pregnancy. In fact, compared to the poorest households, the hazard of becoming pregnant before marriage was 24% lower in the middle class and 65% lower in the households with a high standard of living. Compared to middle-standard households, those with a high standard of living had a lower risk of premarital pregnancy (see confidence intervals in Table 4).

The risk of premarital pregnancy was also higher for young women whose heads of households did not attend school (Table 4). When a head of a household had reached the secondary level, the risk of becoming pregnant was 32% lower, compared to the case where the head of the household had no formal education at all. When he had the level of primary education, this risk was 26% lower, compared with the uneducated one. Women whose heads of households had a primary level of education and whose heads of households had reached the secondary level did not show a significant difference in terms of a risk of pregnancy (Table 4).

The parental presence was associated with an increased risk of getting pregnant, with women who lived with neither their father nor their mother, having less than half the risk of getting pregnant before marriage, as compared to the other women (Table 4). There was no significant difference between those who lived with only one parent and those who lived with both parents (Table 4). There was also no significant difference in the risk of getting pregnant based on the size of the household (Table 4). Regarding the type of neighborhood, women living in formal neighborhoods and those in informal settlements were not different in terms of a risk of premarital pregnancy (Table 4).

With respect to the explanatory power of the cultural inheritance model, the two variables used, that is, ethnicity and religion, had a significant relationship with the risk of getting pregnant before marriage (Table 4). Mossi women had a 36% lower hazard of premarital pregnancy, compared to women from other ethnic groups. Regarding religion, our results indicated a higher risk of pregnancy among Christians as compared to Muslims (Table 4). Christian ladies had a 28% higher hazard of getting pregnant before marriage, all things equal otherwise.

Finally, regarding our control variables (the woman’s age group and her level of education), the results showed that the risk of experiencing any premarital pregnancy was lower among younger women, with women who were 15–19 years old, having a hazard of 63% lower than women aged 20–24 (Table 4). Having a secondary education or above also meant a 31% higher hazard of pregnancy compared to those with a primary education or below (Table 4).

## 5. Discussion

The purpose of this paper was to report findings from a study that sought to understand risk factors of premarital pregnancy in an urban context of West Africa, specifically in Ouagadougou, Burkina Faso. We used a quantitative approach based on longitudinal data, to test the explanatory power of two theories, namely the cultural inheritance model and the social capital model. Longitudinal data have rarely been used to study topics like the one related to the risk factors of premarital pregnancy.

The multivariate results confirmed the explanatory power of the cultural inheritance model, despite the fact that according to Bankole et al. (2013), social taboos that prevail over premarital sexual activity in sub-Saharan African countries tend to fade with the modernization of economies, the increase in the proportion of girls enrolled in school, and the weakening of social control in connection with sexual behavior. The two variables selected (religion, ethnicity) to test the cultural inheritance model have all proved to be significant, and the relationships highlighted were in conformity with our Hypotheses 1 and 2.

In fact, we found that premarital pregnancies were more frequent among Christian adolescents and young women, whereas according to Rwenge (1999), the Christian religion in Africa, which is closely linked to Western values, encourages the use of contraceptive methods to avoid unwanted pregnancies. As it was found in a study conducted by UNFPA (2013) in Burkina Faso, Christian women had a contraceptive prevalence rate that was 1.4 times higher than Muslim women. UNFPA’s study involved married women aged 15–49. Generally speaking, the use of contraception among unmarried young women is rather occasional and dominated by the use of the male condom as suggested by findings of previous work in Burkina Faso (Adohinzin et al., 2016; Sawadogo, 2016). In this case, it is possible that the gap between Christian and Muslim young women in terms of the use of contraception is rather small. Between these two groups, the difference showed by our results in terms of risk of premarital pregnancy may be explained by a higher level of risk-taking among young Christian women, that is, more frequent sexual activity, in comparison with their Muslim counterparts.

The relationship between religious affiliation and sexual activity had already been demonstrated by Okigbo and Speizer (2015) who showed that in five Kenyan cities (Nairobi, Mombasa, Kisumu, Machakos, and Kakamega), unmarried women aged 15–24 who are of the Catholic denomination (vs. Muslims and other Christians) were more likely to have transitioned to first sex at each age even if this did not lead to an earlier transition to first pregnancy.

With regards to ethnicity, the lower risk of premarital pregnancy with Mossi women may mean that out-of-wedlock childbearing in this ethnic group can perhaps more than the other ethnic groups expose women to stigma and sometimes to family exclusion. As it was indicated in the theoretical background, the Mossi cultural values are classified among the most sexually conservative in Burkina Faso (Bonnet, 1988; Taverne, 1999). For instance, in a recent study report, Zongo, Sambiéni, and Paul (2015), comparing Mossi and Fulani in terms of management of pregnancy before marriage, noted that among the Fulani, there was no ban on the girl who contracted a pregnancy, while among the Mossi, there were still cases of punishment like being “out-casted” by the family. But he noted that this form of punishment was rather rare in urban settings (Zongo et al., 2015).

Studies that have tested the cultural inheritance model, particularly the influence of ethnicity on premarital fertility in sub-Saharan Africa, are very rare. A nice attempt is the work of Garenne and Zwang (2006) who used Demographic and Health Survey data from 25 countries to study the variations of premarital fertility by ethnicity in sub-Saharan Africa. Based on these data, they examined a total of 263 ethnic groups and revealed a significant variation in the prevalence of premarital fertility by ethnic group. Their analysis showed that socioeconomic determinants explained about half (57%) of the total variance of the prevalence of premarital fertility, the rest being due to ethnicity, which emphasizes the important role of cultural factors in childbearing before marriage in Africa.

Our results also confirmed two of the five hypotheses in connection to the social capital model (Hypotheses 4 and 6). We found that premarital pregnancy had a negative correlation with the standard of living of the household as well as with the educational level of the head of household, even if the influence of the latter was visible only from the secondary level. By virtue of these two results, we cannot deny the fact that the social capital model has some explanatory power in the context of Ouagadougou. However, we consider this power as partial giving the fact that three of the five hypotheses in connection with this theory were invalidated by our findings.

The relationship with the standard of living of the household was found consistent with qualitative results from Rossier et al. (2013) in Ouagadougou, showing that women from the upper classes were less likely to get pregnant. This influence may be due to the fact that usually women who are not poor have greater access to modern methods of contraception (Rossier et al., 2013). It may also mean that in a context of financial or material precarity, adolescents and young women may deliberately engage in sex with a wealthier partner in order to improve their living conditions as evidenced by Calves (1999) in Yaoundé, Cameroon. In this sense, the influence of the standard of living of the household may also reflect the rational adaptation model. Mazzocchetti (2010) had already highlighted such a rationality in the choice of the partner by young women in Ouagadougou. Drawing on an ethnographical research, she had noticed that young women were divided between an injunction to marry, a desire to find a capable spouse, and a difficult socioeconomic context. Therefore, the exchange of bodies became for many of these women not only a means of escaping poverty but also a means of planning for the future (Mazzocchetti, 2010).

Our finding with the standard of living is contrary to the result found by Emina (2009) in the Democratic Republic of Congo (DRC), where premarital childbearing among women was rather higher in the economically advantaged households. In this country, specifically in the capital city Kinshasa, Djamba (2003) also highlighted a positive relationship between household wealth and premarital sexual activity: girls from poorer families were less likely to engage in premarital sexual intercourse than their counterparts in wealthier families. Djamba’s finding called for other types of explanation: less paternal attention on girls living in wealthier families, great value attached by poor families to their daughters’ chastity in the context of Kinshasa, where “financial capital and female virginity can be considered as exchangeable ‘goods’ in the marriage market” (Djamba, 2003, p.335). Another explanation was that men who usually engage in sexual relationships (especially in case of casual relationships) are more attracted to beautiful girls, and girls from wealthy families are more attractive than those from poor families (Djamba, 2003).

Unexpectedly, the parental presence in the home played a positive influence on the adolescent or young woman’s premarital pregnancy (Hypothesis 7 rejected). This counterintuitive relationship has also been observed in Nairobi, especially on the risk of unwanted pregnancies among young women (Beguy, Mumah, & Gottschalk, 2014). This finding was in line with another study (Kabiru, Beguy, Undie, Zulu, & Ezeh, 2010), which found that a high level of parental supervision was positively associated with a high risk of sexual debut among adolescent girls, which in turn was also associated with the risk of unwanted pregnancies. Excessive parental control, as perceived by adolescent girls, may increase risky sexual behaviors (Rodgers, 1999). In the case of Ouagadougou, our result may also reflect a higher level of awareness of the vulnerability among adolescents and young women in the absence of their parents. For instance, Faure (2014) argued that, beyond the pain of mourning, young adults who have lost a parent might develop a psychological sense of adulthood earlier than their peers. Having lived through hardship and having to be so self-reliant may make young women in these situations more dedicated to ensuring stability and avoiding additional precarity. This could include taking more care to avoid a premarital pregnancy. This relationship (lower premarital pregnancy risk in case of parental absence in the household), which conflicts with the supposed theoretical influence in the social capital model, is a strong result of our study.

The size of the household and the type of neighborhood are the two variables related to the social capital model that showed no relationship with the risk of premarital pregnancy (Hypotheses 3 and 5 rejected). The absence of a relationship between the size of the household and premarital pregnancy in the Ouagadougou HDSS is not therefore consistent with the idea of a dilution of adult attention toward the child in larger families as suggested by Coleman (1988). According to Gassama (2009), the size of the family must be an argument to be contextualized. Large families, which are very common in West Africa, cannot predetermine any disadvantage of children (Gassama, 2009). In fact, African families are often extended to grandparents, aunts and/or uncles, cousins and/or nephews (nieces) in the family. More intra-family relationships may even provide solidarity and enriching social experiences for children and adolescents (Gassama, 2009; Locoh, 1995). These relationships also promote social control (Barou, 2017). As a result, the size of the family does not necessarily contribute to the risk of premarital pregnancies among adolescent or young women.

With regards to our control variables, the positive correlation between the woman’s age and the risk of pregnancy gives credence to the demographic model of premarital fertility, whereby the gap between the onset of the organic sexual need and marriage is positively correlated with the risk of premarital pregnancy. The positive correlation between the woman’s educational level and the risk of premarital pregnancy supports the assumption of a positive link between education and modernism (Cherlin & Riley, 1986), which in turn is correlated with the westernization of values and the weakening of the traditional family system. In this sense, the social disorganization model also applies to Ouagadougou.

## 6. Limitations

Our study had some limitations. Because of the periodicity of data collection in the Ouagadougou HDSS (10 months), some pregnancies terminated earlier by abortions or miscarriages may not have been reported by the respondents. Thus, the estimated pregnancy rates in our analysis were likely to be underestimated. The lack of information on religious beliefs and practices required that we limited our measures to religious affiliation as the proxy for religion. But as we know, religiosity, that is, the level of adherence to religious principles, varies enormously from one person to another within the same religion. Even though we were able to highlight an effect of religion on premarital pregnancy, a measure of religiosity would better capture the impact of religion. This holds true for the ethnic group variable as well. Although we have observed an effect of this variable, we must recognize that at the individual level, one can be of a given ethnic group but has socialized in an environment dominated by the principles of another ethnic group. Considering the dominant culture of the socialization environment could also help refine the impact of the ethnic group.

Finally, it is important to underline that variables used to test the social capital model were few. We only tested this model at the micro level given the limited data available. A complementary qualitative survey would be an important next step to highlighting the effect of the social capital on a larger scale. This survey would also make it possible to better understand the relational context in which a pregnancy occurred. With regards to our proxy of the standard of living, it should be noted that, as in any analysis of poverty, it is possible that some households qualified as poor are able to meet their needs on a permanent basis while other households qualified as nonpoor cannot meet theirs.

## 7. Conclusion

Pregnancies among adolescents and young women before marriage are a major issue in Burkina Faso (AFP, 2016; UNFPA, 2014). For instance, in December 2017, a press article evoked the concerns of the authorities of the Kadiogo Province (in which Ouagadougou is located), faced with an increase in the number of early and unwanted pregnancies in schools (AIB, 2017). In some cases, the management of such pregnancies is done without much difficulty as evidenced by Gastineau (2009) or by Mondain, Delaunay, and Adjamagbo (2009). But in many other cases, it can be challenging, especially if the relationship in which the pregnancy occurred is an occasional one without any serious commitment. The situation may be complicated if the boyfriend lives in precarious conditions or does not accept the paternity of the pregnancy. The possible consequences of such pregnancies are multiple, ranging from girls leaving school early to their family and social exclusion, unsafe clandestine abortions, child abandonment, contested paternity, difficult living conditions (Adjamagbo et Kone, 2013; Bankole et al., 2013; Calves, 2000; Izugbara & Egesa, 2014).

Despite the abovementioned limitation, our study contributes to the knowledge on premarital pregnancy, by identifying some determinants. It succeeded in reaching some findings showing that both the cultural inheritance model and the social capital model have some explanatory power on the risk of becoming pregnant before marriage in Ouagadougou. The control variables used also reflected by their influence, the social disorganization, and demographic models of premarital fertility, even if this study did not aim at specifically testing the explanatory power of those two models.

An implication of the results of this study is the need for a multipronged approach to sexual and reproductive health for young people, which includes combating poverty and inequality, strengthening sexual education, and improving access to contraceptives. For instance, youth friendly sexual and reproductive health services must be available to young people, with convenient hours, accessible locations, and confidential services. Our findings in connection to the social capital model suggest that sexual education should not be solely carried by educators and/or specific programs but should also involve parents more than any other actor. This is not yet the case in the current Burkina Faso’s context where talking to teenage girls about their sexuality is a total discomfort for many parents, as shown by findings from previous studies (Bankole, Biddlecom, Guiella, Singh, & Zulu, 2007; Hien et al., 2012; Some et al., 2012).

In other words, strategies to reduce the number of premarital pregnancies must be based on participatory approaches that incorporate actions at both community and institutional levels, as suggested by the Global Accelerated Action for the Health of Adolescents logical framework (WHO, 2017). For example, it would be beneficial to raise awareness at the community level, taking into account both women and men. Families need to be made aware of the need of their support to prevent adolescent pregnancies. Families must also support these adolescent pregnancies in case they occur. The social and family condemnation of premarital pregnancies in Burkina Faso often leads to a frequent recourse to abortion by adolescent girls, as supported by Bankole et al. (2013).

At the institutional level, centers for social action must be more involved as mediators in the resolution of pregnancy-related conflicts between the two partners or their families. They must also be involved as a moral and financial support to girls who carry unwanted pregnancies and who do not have the means to take care of themselves. In addition, the Ministry in charge of the education of Burkina Faso has been testing student clubs and associations, like student committees designated by their peers to help raise awareness on various issues including reproductive health (Zongo et al., 2015). The action of these clubs must be reinforced through financial support, trainings, and seminars, and their members must be provided with the knowledge and skills needed to efficiently act toward the prevention of premarital pregnancies and sexually transmitted diseases.
